# Interactions between cauliflower and *Rhizoctonia *anastomosis groups with different levels of aggressiveness

**DOI:** 10.1186/1471-2229-9-95

**Published:** 2009-07-21

**Authors:** Joke Pannecoucque, Monica Höfte

**Affiliations:** 1 Laboratory of Phytopathology, Faculty of Bioscience Engineering, Ghent University, Coupure Links, 653, B-9000 Gent, Belgium

## Abstract

**Background:**

The soil borne fungus *Rhizoctonia *is one of the most important plant pathogenic fungi, with a wide host range and worldwide distribution. In cauliflower (*Brassica oleracea *var. *botrytis*), several anastomosis groups (AGs) including both multinucleate *R. solani *and binucleate *Rhizoctonia *species have been identified showing different levels of aggressiveness. The infection and colonization process of *Rhizoctonia *during pathogenic interactions is well described. In contrast, insights into processes during interactions with weak aggressive or non-pathogenic isolates are limited. In this study the interaction of cauliflower with seven *R. solani *AGs and one binucleate *Rhizoctonia *AG differing in aggressiveness, was compared. Using microscopic and histopathological techniques, the early steps of the infection process, the colonization process and several host responses were studied.

**Results:**

For aggressive *Rhizoctonia *AGs (*R. solani *AG 1-1B, AG 1-1C, AG 2-1, AG 2-2 IIIb and AG 4 HGII), a higher developmental rate was detected for several steps of the infection process, including directed growth along anticlinal cell walls and formation of T-shaped branches, infection cushion formation and stomatal penetration. Weak or non-aggressive AGs (*R. solani *AG 5, AG 3 and binucleate *Rhizoctonia *AG K) required more time, notwithstanding all AGs were able to penetrate cauliflower hypocotyls. Histopathological observations indicated that *Rhizoctonia *AGs provoked differential host responses and pectin degradation. We demonstrated the pronounced deposition of phenolic compounds and callose against weak and non-aggressive AGs which resulted in a delay or complete block of the host colonization. Degradation of pectic compounds was observed for all pathogenic AGs, except for AG 2-2 IIIb. Ranking the AGs based on infection rate, level of induced host responses and pectin degradation revealed a strong correlation with the disease severity caused by the AGs.

**Conclusion:**

The differences in aggressiveness towards cauliflower observed among *Rhizoctonia *AGs correlated with the infection rate, induction of host defence responses and pectin breakdown. All *Rhizoctonia *AGs studied penetrated the plant tissue, indicating all constitutive barriers of cauliflower were defeated and differences in aggressiveness were caused by inducible defence responses, including cell wall fortifications with phenolic compounds and callose.

## Background

During the interaction with pathogens, plants that recognize the intruder will respond with an impressive battery of defence mechanisms. Both structural and chemical barriers are involved which can be constitutive and/or inducible. Upon pathogen detection, the activated defence responses in the plant may involve the rapid production of reactive oxygen species, hypersensitive response (HR) at the site of infection, strengthening of the cell wall by oxidative cross-linking of cell wall components, apposition of callose or phenolic compounds and the production of phytoalexins and pathogenesis-related proteins [1-4]. Typically, these responses can be very localised and microscopic observations seem to be the most appropriate method for investigation [5]. Before succeeding in causing disease, the pathogen must penetrate the plant and overcome these obstacles; this consequently explains why plant pathogens colonize only a narrow range of hosts.

Among members of the fungal genus *Rhizoctonia*, the ability to cause disease is highly variable and depending on the host plant. *Rhizoctonia *comprises both multinucleate and binucleate species which are further divided into anastomosis groups (AGs). Currently, the multinucleate species *R. solani *(teleomorph: *Thanatephorus cucumeris*) contains 13 AGs (AG 1 – AG 13) [6], while binucleate *Rhizoctonia *spp. (teleomorph: *Ceratobasidium *spp.) are divided into 16 AGs (AG A – AG I, AG K, AG L, AG O – AG S) [7]. Within the same AG, isolates may possess similar characteristics such as type of disease symptoms caused and host preference [8]. In addition, for several host-pathogen interactions, isolates of the same AG have comparable levels of aggressiveness.

During pathogenic interactions of *Rhizoctonia *isolates with several host plants, the early steps of the infection process appear to be very similar independent of AG or host [9]. *Rhizoctonia *hyphae adhere to the plant surface and soon this is followed by directed growth along the anticlinal epidermal cell walls and formation of T-shaped branches. Infection cushions are formed from which infection pegs penetrate the plant tissue. During some interactions, especially for isolates obtained from leaves, the formation of infection cushions is rather exceptional and the pathogen will form lobate appressoria or penetrate the plant through stomata [10]. Further penetration and colonization have been associated with the enzymatic degradation of the host tissue, including pectic substances and cellulose [11,12].

In contrast with the well-studied and generally accepted situation for pathogenic interactions, insight into the processes during non-pathogenic interactions is still largely missing. Keijer [9] reported two distinct observations during the early steps of an incompatible infection process. Stomatal penetration of *R. solani *AG 2 BI resulted in hypersensitive-like lesions on cauliflower stems, while AG 3 could not adhere to the cauliflower surface nor proceed to further steps of the infection process. Resistance of plant species or cultivars to *Rhizoctonia *has also been associated with cuticle thickness [13], wax deposits [14], accumulation of calcium [15], and inhibition of pectin degrading enzymes [16]. Jabaji-Hare et al. [17] reported the induction of various cell wall compounds, such as suberin, pectic substances and phenolic compounds, during the interaction of bean with a non-pathogenic binucleate isolate. All these phenomena have been observed for specific AGs on specific hosts and a generally accepted situation for non-pathogenic interactions has not been described.

When studying the infection process of *Rhizoctonia*, researchers have always focussed on the differences between susceptible and resistant cultivars inoculated with the same isolate [14,18-20]. Until now, no in depth study has been carried out to compare the infection and colonization process and the host responses induced in the same plant cultivar by different *Rhizoctonia *AGs reflecting variation in aggressiveness.

Previously, we reported the importance of seven *R. solani *AGs and one binucleate *Rhizoctonia *AG possessing different levels of aggressiveness, in association with Belgian cauliflower production [21]. However, until now it is not clear at which stages during the infection process of cauliflower differences in aggressiveness appear. Here, in an attempt to gain more insights into the processes underlying pathogenic and non-pathogenic *Rhizoctonia*-cauliflower interactions, we investigate the infection process, the host colonization and the induced defence reactions of those eight *Rhizoctonia *AGs. In this study, we provide evidence that *Rhizoctonia *AGs differ in the developmental rate of the infection and colonization process and provoke differential host responses and pectin degradation. Moreover, we show a strong correlation between these microscopic observations and the disease severity caused by the AGs. Interestingly, we observed that all *Rhizoctonia *AGs are able to enter cauliflower hypocotyls, although weak and non-aggressive AGs require more time and form less infection cushions. Studying histopathological sections, we demonstrate the pronounced deposition of phenolic compounds and callose against weak and non-aggressive AGs which slowed down or stopped the fungal growth.

## Methods

### Fungal isolates

*Rhizoctonia *isolates were selected from the collection of Pannecoucque et al. [21] based on AG and aggressiveness. Since isolates within the same AG had the same level of aggressiveness [21], one representative isolate per AG was selected. Seven *R. solani *isolates were included representing AG 1-1B (BK004-2-1), AG 1-1C (BK010-1-1), AG 2-1 (BK001-1-1), AG 2-2 IIIb (M001-1-1) and AG 4 HGII (BK004-1-1) which were previously considered to be aggressive towards cauliflower; AG 3 (BK006-2-1), AG 5 (BK003-1-3) and one binucleate *Rhizoctonia *isolate of AG K (BK005-1-1) which were identified as weak or non-aggressive. All isolates were obtained from Belgian cauliflower fields, except isolate M001-1-1 which was isolated from maize. Isolates were maintained at room temperature on PDA and in the dark.

### Cauliflower plants and growth conditions

All experiments were carried out using plants of *Brassica oleracea *var. *botrytis *cv. Clapton (Syngenta Seeds, the Netherlands).

To study early steps of the infection process and degradation of pectic compounds, cauliflower plants were grown *in vitro *under sterile conditions as earlier described [21]. Briefly, cauliflower seeds were surface-sterilized in 0.5% NaOCl-solution containing 0.01% Tween 20 and rinsed twice with sterile demineralized water. Gamborg B5 medium (Gamborg B5 basal salt mixture; Labconsult) solidified with 1% (w/v) agar and enriched with vitamins (Gamborg B5 vitamin mixture; Labconsult) was poured into square Petri dishes (12 × 12 cm; Novolab) in which the *in vitro *plants were grown. Six surface-sterilized cauliflower seeds were placed at equal intervals on the agar layer. The Petri dishes were sealed with Parafilm (Novolab) and incubated in the dark at 21°C for 2 days to allow seed germination. Afterwards, the lower halves of the Petri dishes were wrapped into aluminium foil to protect the roots from the light and the dishes were placed during 8 days in an upright position at an angle of 60° in a growth chamber (21°C, 16 h photoperiod).

For aggressiveness assays and the histopathological study, cauliflower plants were grown in the growth chamber (21°C, 16 h photoperiod, 60–70% relative humidity). Cauliflower seeds were sown in trays (22 × 15 × 6 cm) filled with commercial non-sterile potting soil (Structural; Snebbout, Kaprijke, Belgium) and regularly watered until three true leaves had developed.

### *In vitro *assay for the study of the initial steps of the interaction process

Ten-day-old sterile *in vitro *grown cauliflower plantlets were inoculated with different *Rhizoctonia *AGs. Agar discs (diameter 5 mm) overgrown with *Rhizoctonia *mycelium were taken from 4-day-old PDA plates and placed in the square Petri dishes beside each cauliflower hypocotyl. Plant stems were sampled with an interval of 6 hours, starting at 0 hours post inoculation (hpi) and ending at 120 hpi, and placed in 100% ethanol. After fixation and chlorophyll removal, *Rhizoctonia *hyphae were stained in 0.1% (w/v) trypan blue in 10% (v/v) acetic acid for 10 min and rinsed in distilled water to remove excess stain. For each time point and each AG, at least five plant stems were studied using light microscopy. To improve picture quality, some samples were longitudinally hand-cut using a razor blade. The experiment was repeated once.

### Production and purification of pectin degrading enzymes

Extracellular pectin degrading enzyme production was stimulated using two types of liquid medium. The first medium was prepared according to Schneider et al. [22] and contained 1% citrus pectin (Sigma-Aldrich). In the second medium, the pectin was replaced by 1% of cauliflower cell walls prepared as described by Bugbee [23]. *Rhizoctonia *isolates were grown in the dark on a rotary shaker at 100 rpm. After 10 days of incubation, liquid cultures were filtered through Whatman No. 1 filter paper, centrifuged at 15 000 g for 15 min and filter sterilized through 33 mm Millex Filter Units with a filter pore size of 0.22 μm (Millipore, Brussels, Belgium). Sterile culture filtrates were added to 32-well-plates containing in each well a sterile cauliflower cotyledon excised from a 10-day-old *in vitro *grown cauliflower plantlet. After 24 hours, cotyledons were removed from the culture filtrates and transferred to 100% ethanol. After fixation and chlorophyll removal, cotyledons were stained using 0.005% ruthenium red in water for 30 min which stains pectic compounds red [24] or with 0.05% toluidine blue in citrate/citric acid buffer (50 mM, pH 3.5) for 10 min which stains polyphenols green to blue-green and pectic compounds pink to purple [25]. All samples were studied using light microscopy. The experiment was repeated twice.

### Aggressiveness assays

Cauliflower plants with three leaves were transplanted to pots (diameter 9 cm, height 9 cm) containing non-sterile potting soil (Structural; Snebbout, Kaprijke, Belgium). *Rhizoctonia *inoculum was produced on wheat kernels which were soaked for 24 h in tap water [26]. The kernels were autoclaved twice on two consecutive days and inoculated with three PDA discs (diameter 7 mm) of 4-day-old *Rhizoctonia *cultures. The kernels were incubated for 10 days at room temperature in the dark and shaken every 3–4 days. Plants were artificially inoculated with *Rhizoctonia *by placing three infected wheat kernels around each plant, 2 cm away from the plant and 2 cm below the soil surface. Disease symptoms were evaluated at 6 days post inoculation for all AGs. Highly aggressive isolates (AG 1-1B, AG 1-1C, AG 2-1, AG 2-2 and AG 4 HGII) were also evaluated at an earlier time point (3 days post inoculation) and weak or non-aggressive isolates (AG 3, AG 5 and AG K) at a later time point (12 days post inoculation). An evaluation scale based on phenotypical observations was used: 0 = healthy, no symptoms; 1 = HR-like spots or resistant reaction; 2 = HR-like spots + small susceptible reaction (< 2 mm); 3 = small susceptible reaction (< 2 mm) and 4 = large susceptible reaction (> 2 mm). The experiment was carried out with 10 plants per AG at each time point and was repeated once.

### Histopathological analysis of the *Rhizoctonia*-cauliflower interaction

For histological observations, pieces of cauliflower hypocotyls (5 mm in length) were excised from inoculated plants and control plants. At each sampling time (3, 6 and 12 days post inoculation), the hypocotyls of 10 inoculated plants per *Rhizoctonia *AG and 3 control plants were sampled. Tissue samples were fixed overnight at 4°C in 50 mM Na phosphate buffer (pH 7.2) containing 4% paraformaldehyde and 1% glutaraldehyde and dehydrated at room temperature in a graded series of ethanol concentrations (30, 50, 70, 85, 96 and 100%) for at least 2 h for each concentration. After dehydration, samples were infiltrated at 4°C in 1:1 and 0:1 (vol/vol) ethanol/Technovit 7100 infiltration solution and embedded in plastic moulds using Technovit 7100 histo-embedding medium (Heraeus Kulzer, Wehrheim, Germany) according to the manufacturer's instructions. The plastic moulds were closed and polymerisation started at room temperature for 1 h, followed by an overnight incubation at 37°C. Embedded tissue was sectioned into transversal semi-thin sections (2 μm) with a Leica RM2265 motorised rotary microtome (Leica Microsystems, Nussloch, Germany) equipped with a glass knife and sections were mounted on microscope glass slides. To each sample, differential staining procedures were applied. Staining in 1% toluidine blue for 3 min yielded a good differentiation between plant cells and fungal hyphae and was used to study the colonization process. To visualize pectic compounds, sections were stained in 0.005% ruthenium red for 5 min [24]; to visualize cell wall fortifications with phenolic compounds, a solution of 0.01% safranin O in 50% ethanol was used and samples were stained for 3 min [27]. Sections were cover slipped with DPX neutral mounting medium (containing distrene 80 – dibutylphthalate – xylene; Klinipath, Belgium) before examination under light microscopy. For the visualization of callose, sections were stained with 0.05% aniline blue in 0.067 M K_2_HPO_4 _at pH 9.0 [28]. The stain solution was prepared at least two hours prior to use; samples were mounted in DPX and examined using fluorescence microscopy.

### Microscopic observations and statistical analyses

All microscopic observations were performed with an Olympus BX51 microscope (Olympus, Aartselaar, Belgium) equipped for fluorescence microscopy with a UV filter (330–385 nm excitation filter, DM 400 dichroic beam splitter and BA420 long-pass filter). Digital images were acquired using an Olympus Color View II camera (Aartselaar, Belgium) and further processed with Olympus analySIS cell^F software (Olympus Soft Imaging Solutions, Münster, Germany).

Statistical analyses were carried out using the software package SPSS 15.0 for Windows. Because the categorical data did not fulfil the assumptions of normal distribution and homogeneity of variances, non-parametric tests were performed including Kruskal-Wallis and Mann-Whitney comparisons (p = 0.05) and Spearman's rho correlation (p = 0.01).

## Results

### Initial steps during the infection process

**Figure 1:**
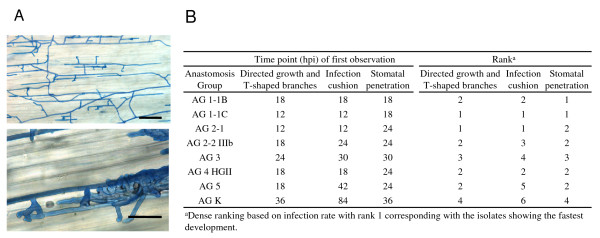
**Initial steps during the infection process of cauliflower with seven *R. solani *AGs and one binucleate *Rhizoctonia *AG**. **A**, Microscopic observations of trypan blue stained *Rhizoctonia *hyphae growing along anticlinal cell walls of cauliflower and branching in T-shaped angles (upper photograph) and formation of infection cushions (lower photograph). Scale bars = 100 μm. **B**, Time point (hours post inoculation) of first observation of directed growth of *Rhizoctonia *hyphae along anticlinal cell walls and formation of T-shaped branches, formation of infection cushions and penetration through stomata.

The initial steps in the infection process of cauliflower seedlings by seven isolates of different *R. solani *AGs and one isolate of binucleate *Rhizoctonia *AG K were compared. At a 6 hours interval, stems of cauliflower plantlets were examined for the presence of adhered hyphae, directed growth along anticlinal epidermal cell walls and T-shaped branched hyphae, infection cushions and penetration sites through stomata (Fig. 1). At 12 hpi all *Rhizoctonia *AGs were adhered to the stem surface of cauliflower, since hyphae were not removed by washing the stems under tap water and fixation in ethanol. From this time point onward, the developmental rate of the infection process differed among the AGs. Formation of T-shaped branches and directed growth was first observed for the pathogenic isolates of AG 1-1C and AG 2-1 at 12 hpi, followed by AG 1-1B, AG 2-2 IIIb, AG 4 HGII and AG 5 isolates at 18 hpi. For the isolate of AG 3, this growth pattern was evident at 24 hpi and for the non-pathogenic binucleate isolate of AG K at 36 hpi. The rate of infection cushion formation followed the same tendency and these structures were first detected for the pathogenic isolates of AG 1-1C and AG 2-1 at 12 hpi, followed by the isolates of AG 1-1B and AG 4 HGII at 18 hpi; the isolate of AG 2-2 IIIb developed infections cushions at 24 hpi. For the isolates of AG 3 and AG 5, infection cushions were observed at 30 hpi and 42 hpi, respectively, while the binucleate AG K isolate formed very little infection cushions of which the first were noticed at only 84 hpi. Stomatal penetration seemed to occur mostly by coincidence. Hyphae did not seem to be attracted towards stomata, since they frequently grew along without penetration. For the majority of the AGs, stomatal penetration was observed at 24 hpi. In contrast, for the isolates of AG 1-1B and AG 1-1C stomatal penetration was more abundant and was already observed at 18 hpi. The isolates of AG 3 and AG K had the slowest stomatal penetration at 30 hpi and 36 hpi, respectively.

### Role of pectin degrading enzymes in pathogenicity

**Figure 2:**
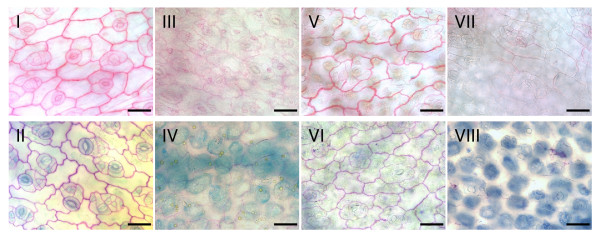
**Degradation of cauliflower cell walls by extracellular produced pectic enzymes of seven *R. solani *AGs and one binucleate *Rhizoctonia *AG**. Microscopic observations of pectic components in cauliflower cotyledones visualized with ruthenium red (I, III, V & VII) and toluidine blue (II, IV, VI & VIII) staining after 24 h incubation in sterile culture filtrate of liquid pectin medium inoculated with a sterile PDA plug as control treatment (I & II), inoculated with *R. solani *AG 3 (III & IV), inoculated with *R. solani *AG 4 HGII (V & VI), after 24 h incubation in sterile culture filtrate of liquid cauliflower medium inoculated with *R. solani *AG 4 HGII (VII & VIII). Scale bars = 50 μm.

Under the *in vitro *conditions tested in this study, all *Rhizoctonia *AGs were capable of producing pectic enzymes which reduced the staining intensity of ruthenium red and toluidine blue (Fig. 2). Compared with the cotyledons of the control treatment, for which both staining protocols resulted in a specific coloration of the cell walls, the cotyledons incubated in the culture filtrate of the eight *Rhizoctonia *AGs showed a clear degradation of the cell walls, including degradation of pectic compounds as indicated by the absence of ruthenium red staining and pink or purple staining by toluidine blue. No differences were observed between isolates of pathogenic and non-pathogenic AGs, suggesting all isolates produced pectinolytic enzymes that could degrade pectin of cauliflower. Because the extracellular production of pectin degrading enzymes depends upon the growth medium [29], two different liquid media were tested; one which contained citrus pectin and one with cauliflower cell walls. Only the isolate of AG 4 HGII yielded different results for the two media. The culture filtrate of the AG 4 HGII isolate grown on pectin medium did not cause a degradation of pectin, while for the culture filtrate of the cauliflower cell wall medium a clear degradation of the cotyledonous cell walls was detected.

### Aggressiveness assays

Symptom evaluation at 3 dpi resulted for the aggressive isolates of AG 1-1B, AG 1-1C, AG 2-1, AG 2-2 IIIb and AG 4 HGII in a disease severity index (DSI) exceeding 3, indicating all symptoms observed showed a susceptible reaction zone (Table 1). For these AGs, resistant reactions were never observed. At 6 dpi, all AGs were evaluated and only susceptible reactions were observed for the aggressive isolates of AG 1-1B, AG 1-1C, AG 2-1, AG 2-2 IIIb and AG 4 HGII. For the weak aggressive isolates of AG 5 and AG 3, the majority part of the plants showed HR-like lesions, although some susceptible reactions were also observed (DSI = 1.6 and 1.4 respectively). Infection with the binucleate isolate of AG K only resulted in HR-like lesions (DSI = 0.9). To check whether the symptoms caused by the weak and non-aggressive isolates would shift towards susceptible reactions, an extra time point at 12 dpi was included. This was the case for the AG 5 isolate, for which at 12 dpi all lesions were from the susceptible type and HR-like lesions were no longer observed (DSI = 3.8). For the AG 3 isolate, a higher proportion of plants showed small susceptible reactions combined with HR-like lesions (DSI = 1.6) and in the case of AG K, all plants showed HR-like lesions, while susceptible reactions were absent (DSI = 1).

**Table 1:** Disease severity index and average rank of seven different *R. solani *AGs and one binucleate *Rhizoctonia *AG

	Disease severity index						
		
Anastomosis Group*	3dpi		6dpi		12 dpi		Average rank
				
AG 1-1B	3.3	ab	3.7	ab	nd		1.5
AG 1-1C	3.6	a	3.9	a	nd		1.0
AG 2-1	3.2	ab	3.6	ab	nd		1.2
AG 2-2 IIIb	3.0	b	3.4	b	nd		2.3
AG 3	nd		1.4	c	1.6	b	3.8
AG 4 HGII	3.3	ab	3.6	ab	nd		2.2
AG 5	nd		1.6	c	3.8	a	3.2
AG K	nd		0.9	d	1.0	c	5.0

Cauliflower plants were grown in potting soil and possessed three true leaves at the time of artificial inoculation with wheat kernels colonized by *Rhizoctonia*. Evaluation was performed at three different time points using a 0-to-4 scale: 0 = healthy, no symptoms; 1 = HR-like spots or resistant lesions; 2 = HR-like spots + small susceptible reactions (< 2 mm); 3 = small susceptible reactions (< 2 mm) and 4 = large susceptible reactions (> 2 mm). Statistical analysis was performed on pooled data from two experiments, because interaction between AG and experiment was not significant and variations were homogeneous. Different letters indicate statistically significant differences between AGs according to non-parametric Kruskal-Wallis and Mann-Whitney tests (α = 0.05). *Negative significant correlation between average rank and disease severity index at 6 dpi according to Spearman's rho coefficient of -0.958 (p = 0.01). nd = not determined

### Histopathological observations

**Figure 3:**
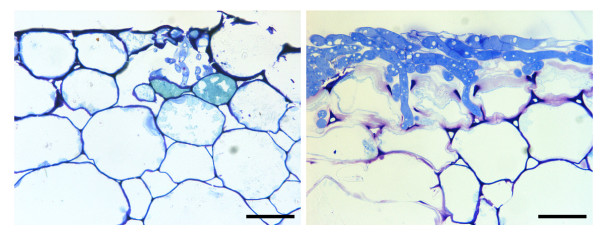
**Toluidine blue staining of transversal sections of cauliflower hypocotyls**. Stomatal penetration at 6 dpi by binucleate *Rhizoctonia *AG K and toluidine blue positive granulation of some adjacent cells (left). Penetration underneath an infection cushion at 3 dpi by *R. solani *AG 2-1 (right). Scale bars = 50 μm.

**Figure 4:**
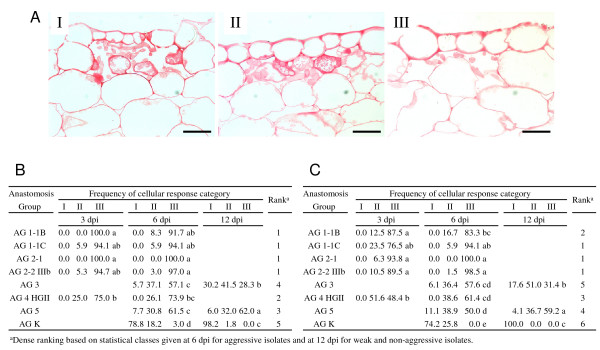
**Safranin O and aniline blue staining of cauliflower hypocotyl cells after infection by seven different *R. solani *AGs and one binucleate *Rhizoctonia *AG**. **A**, Cellular responses observed with safranin O and aniline blue staining were classified into three categories; photographs I-III depict representative examples. (I) *Rhizoctonia *hyphae are completely surrounded by cells fortified with safranin O positive material located in the cell walls or in granules observed in the cytoplasma, restricting further fungal growth. (II) Fortification of cell walls and presence of safranin O positive granules in the cytoplasma is observed for some adjacent cells, although colonization by *Rhizoctonia *hyphae is not stopped. (III) Absence of safranin O positive host responses in cells neighbouring *Rhizoctonia *hyphae. Scale bars = 50 μm. **B**, Frequency distribution of cellular response categories at 3, 6 and 12 dpi for different *Rhizoctonia *AGs. The three values within each cell represent the relative proportion of interaction sites designated as type I, II and III as detected after safranin O staining, respectively. **C**, Frequency distribution of cellular response categories at 3, 6 and 12 dpi for different *Rhizoctonia *AGs. The three values within each cell represent the relative proportion of interaction sites designated as type I, II and III as detected after aniline blue staining, respectively. At each time point, at least 50 interaction sites per AG were studied originating from 10 different cauliflower hypocotyls. Within one column, values followed by the same letter are not significantly different according to Kruskal-Wallis and Mann-Whitney tests (α = 0.05).

**Figure 5:**
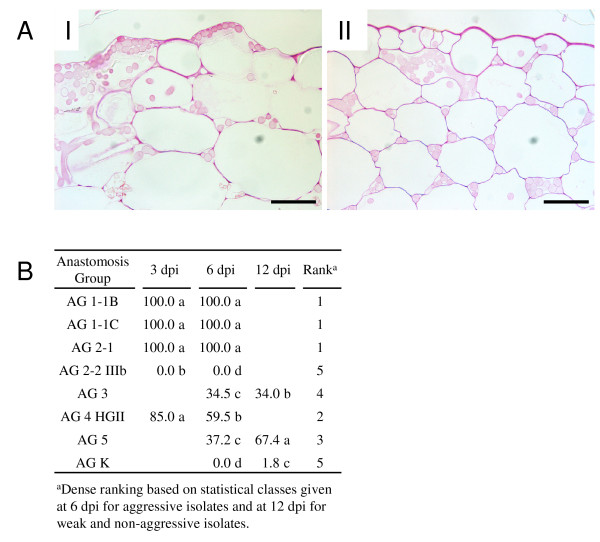
**Ruthenium red staining of cauliflower hypocotyl cells after infection by seven different *R. solani *AGs and one binucleate *Rhizoctonia *AG**. **A**, Cellular responses were classified into two categories (I) Representative example of pectin breakdown as indicated by faint red colour. (II) Uniform red stain of the cell walls indicating absence of pectin breakdown as observed during the interaction with *R. solani *AG 2-2 IIIb. Scale bars = 50 μm. **B**, Relative proportion of interaction sites at which pectin degradation is observed at 3, 6 and 12 dpi during the interaction with different *Rhizoctonia *AGs. At each time point, at least 50 interaction sites per AG were studied originating from 10 different cauliflower hypocotyls. Within one column, values followed by the same letter are not significantly different according to Kruskal-Wallis and Mann-Whitney tests (α = 0.05).

For the aggressive isolates (AG 1-1B, AG 1-1C, AG 2-1, AG 2-2 and AG 4 HGII) samples from 3 and 6 dpi were studied, while the weak and non-aggressive isolates (AG 3, AG 5 and AG K) were studied at 6 and 12 dpi. Penetration of epidermal cells by fungal hyphae occurred both by stomatal penetration and formation of infection cushions under which several penetrating hyphae were observed (Fig. 3). Hyphal penetration was found to be associated with different levels of cell wall modifications. For safranin O and aniline blue stain, cellular responses were classified into three distinct categories (Fig. 4A). In type I and type II, cell wall fortifications were detected at penetration sites of *Rhizoctonia*. In the case of type I, hyphae were completely surrounded by fortified cell walls, thereby restricting further colonization of the host tissue; whereas for type II cell wall depositions were detected although they could not stop the fungal growth and hyphae were observed beyond the fortified cell walls. Type III reactions, on the other hand, were characterized by the absence of cell wall depositions. Staining of the sections with ruthenium red coloured the pectic compounds red. At several interaction sites, pectic compounds were degraded as indicated by the absence of the red stain (Fig. 5A). An overview of the quantitative analysis of the host cell wall responses observed at the interaction sites of the eight *Rhizoctonia *AGs obtained with the three different stains is presented in Figures 4B, 4C and 5B. The majority of the type I and type II reaction sites was, besides the wall thickening, also associated with granulation of the cytoplasm in neighbouring cortical cells. These granules probably contain phenolic compounds since they stained with toluidine blue and safranin O. Eventually, these cortical cells crumpled and collapsed; all these reactions are consistent with a hypersensitive response [30].

Sections of cauliflower stems infected with AG 1-1B, AG 1-1C, AG 2-1, AG 2-2 IIIb and AG 4 HGII stained with toluidine blue showed the abundant and early formation of infection cushions and penetration pegs, resulting in a complete colonization of the cortical cells and vascular tissue at 3 dpi. For the isolates of AG 3 and AG 5, colonization occurred slower and only at 12 dpi hyphae of AG 5 were detected in all parts of the cortex and in the vascular tissue. At that time, hyphae of AG 3 also colonized the cortex and the vascular tissue, although to a lesser extent. The only isolate that was unable to colonize the cauliflower cortex was the binucleate AG K isolate; penetrating hyphae of this AG were limited to substomatal cavities or the first cortical cell layers underneath the penetration site.

Results obtained for the safranin O stain and the aniline blue stain appeared to be very similar (Figs. 4B &4C). For the majority of the interactions at 3 and 6 dpi, infection with AG 1-1B, AG 1-1C, AG 2-1 and AG 2-2 IIIb did not result in the deposition of phenolic compounds or callose as shown by the high percentage of type III interactions. These isolates were closely followed by the isolate of AG 4 HGII for which at 3 and 6 dpi approximately 75% of the interactions were classified as type III for the safranin O stain and 48.4% at 3 dpi increasing to 61.4% at 6 dpi of type III interactions for the aniline blue stain. Between the isolates of AG 3 and AG 5, no significant differences were found at 6 dpi. However, for the isolate of AG 3 at 12 dpi a higher percentage of interactions exhibit type I reactions for safranin O stain (30.2%) and aniline blue stain (17.6%) compared to the AG 5 isolate (6.0% and 4.1%, respectively). The highest induction of phenolic compounds and callose deposition was observed for the binucleate isolate of AG K and at 12 dpi all sites of attempted pathogen entry were associated with an increase in safranin O and aniline blue staining intensity.

Pectin breakdown, studied by ruthenium red staining, was already observed at 3 dpi for all the interaction sites of AG 1-1B, AG 1-1C and AG 2-1 (Fig. 5B). For AG 4 HGII, the majority of the interaction sites also showed pectin degradation. At 6 dpi, around one third of the interaction sites of AG 3 and AG 5 were associated with a fainter ruthenium red staining, although at 12 dpi significantly more pectin breakdown was detected for the AG 5 isolate. For the isolates of AG 2-2 IIIb and AG K, no or only a very low pectin degradation was observed.

### Ranking of AGs and correlation with disease severity

To summarize the results obtained during this research, a ranking was created for the eight *Rhizoctonia *AGs. Following criteria for ranking were included: directed growth, infection cushion formation, stomatal penetration, absence of phenolic compound deposition, absence of callose deposition and pectin breakdown. The first three criteria, collectively referred to as infection rate, were ranked based on the developmental rate of the infection process with rank 1 corresponding with the isolates showing the fastest development (Fig 1B). The other three criteria dealing with the level of induced defence responses and pectin degradation were ranked based on the statistical classes given at 6 dpi for the aggressive isolates and at 12 dpi for the weak and non-aggressive isolates (Figs. 4B, 4C &5B). Based on the average ranking, isolates were ordered starting from AG 1-1C to AG 2-1, AG 1-1B, AG 4 HGII, AG 2-2 IIIb, AG 5, AG 3 and ending with AG K (Table 1). Moreover, a significant negative correlation (p = 0.01) was found between the average ranking and the DSI caused by the different AGs. The Spearman's rho coefficient equals to -0.958, which should be interpreted as the first ranked isolates corresponding with the highest DSI and the isolate with the highest rank corresponding with the lowest DSI.

## Discussion

Although several papers have already been dedicated to the penetration and colonization process of *Rhizoctonia*, the mechanisms involved in the interaction with weak or non-aggressive isolates remain poorly understood. Therefore, a study to compare the interaction between cauliflower and eight *Rhizoctonia *AGs with different levels of aggressiveness was performed. Our observations indicated striking differences among *Rhizoctonia *AGs during the early stages of the infection and colonization process and in the nature and extent of host responses. Moreover, a highly significant correlation was found between disease severity rating and ranking of the AGs based on microscopic observations of the infection process, the level of defence responses and the grade of pectin breakdown.

The pathogenic cauliflower-*Rhizoctonia *interaction, as observed for the first ranked isolates of AG 1-1C, AG 2-1 and AG 1-1B, closely followed by AG 4 HGII, was characterized by a high rate of directed growth, formation of infection cushions and stomatal penetration accompanied with the absence of defence responses and a strong degradation of pectin. The early observation of the different steps in the infection process is in concordance with previous studies concerning pathogenic *Rhizoctonia *AGs on several hosts [9,18,31,32] and the faster and more abundant stomatal penetration of the AG 1-1B and AG 1-1C isolates is probably correlated with the aerial nature of these AGs [33], since isolates from foliage have been reported to penetrate stomata more frequently [10]. During these pathogenic interactions, pectin degrading enzymes seemed important and diffused ahead of the fungus, as pathogen ingress was coupled with extensive host cell deformation and pectin breakdown at locations not in direct contact with hyphae. For many plant pathogens, including *Rhizoctonia*, the role of pectin degrading enzymes in plant cell wall degradation is well established [11,12,23,34]. Pectin degradation of the plant cell wall plays a crucial role in pathogen spread and providing nutriments to the pathogen and therefore, pectin degrading enzymes are potentially important for pathogenicity [35,36].

The small decrease in disease severity, observed for the following ranked isolate of *R. solani *AG 2-2 IIIb, can be ascribed to the later formation of infection cushions and to the remarkable disability to degrade pectic compounds. Notwithstanding pectin degrading enzymes were produced during the *in vitro *experiments and pectic compounds of cauliflower cotyledons were degraded after incubation in the culture filtrate, the degradation of pectin was never observed during the histopathological experiments. A conceivable explanation might involve the different composition of pectin present in different plant parts, such as cotyledons and hypocotyls [37], resulting in a different susceptibility to degradation by the enzymes produced by the AG 2-2 IIIb isolate. Another possibility suggests the involvement of a plant response leading to the production of plant protein inhibitors which prevent cell wall degradation and retard fungal growth and colonization [38]. The slower rate of disease development observed for this isolate further supports this hypothesis. Inhibitory activity of pectin degrading enzymes by plant proteins is considered a part of the plants' immune system and depends on the specific recognition of the pathogen [39]. In the case of *R. solani *AG 2-2, a protein inhibiting pectin lyase activity in sugar beet has already been described [16]. From this point of view, the specific recognition of *R. solani *AG 2-2 IIIb by cauliflower might explain why infection cushion formation and disease development was slower and why this AG was never found in association with cauliflower under field conditions [21]. However, despite the inability to degrade pectic components from the cell wall of cauliflower as observed in this study, AG 2-2 IIIb is generally considered aggressive towards *Brassica *crops [21,40] and as a consequence, during the interaction with cauliflower pectin degrading enzymes are not considered essential for the pathogenicity of this AG.

A slower development of the infection process coinciding with the induction of plant defence responses and a lower level of pectin breakdown was detected for the weak aggressive isolates of *R. solani *AG 5 and AG 3 which ranked next. Possibly the later penetration of the plant tissue allows the plant to build up a defence reaction, as observed by the deposition of phenolic compounds and callose. This defence reaction was more pronounced for AG 3 compared to AG 5. Furthermore, the frequently observed pectin degradation by the AG 5 isolate might help the fungus to overcome the defence responses and to colonize the plant tissue, resulting in a significantly higher disease severity at 12 dpi for AG 5 compared to AG 3. During this study the experimental conditions were in favour of the pathogen, because a relatively high infection pressure was used towards young cauliflower plants. This might explain why during our experiments isolates of AG 5 and AG 3 could provoke such high levels of damage and colonize the complete hypocotyl; while under natural conditions, these AGs are considered not aggressive towards cauliflower [21]. Probably, under field conditions the plant's defence reactions are sufficient to arrest fungal colonization.

A non-pathogenic interaction was identified for the last ranked isolate of binucleate *Rhizoctonia *AG K and was typified by a slow infection rate, resulting in only few infection cushions formed. Contrastingly, Keijer et al. [8] reported that non-pathogenic isolates could not adhere to the plant surface preventing further formation of infection structures. Here, we corroborated the penetration of cauliflower by AG K through stomata and infection cushion formation, indicating the passive defence barriers present in cauliflower can be overcome by this non-pathogenic *Rhizoctonia *AG. Furthermore, a very strong induction of phenolic compounds and callose was observed in association with the absence of pectin breakdown. However, extracellular pectinolytic enzymes produced by AG K could degrade the pectin present in cauliflower cotyledons and the lack in pectin breakdown, as observed during the histopathological experiments, is probably due to the restriction of the fungal growth by the local deposition of cell wall components. Deposition of cell wall fortifications, is a widely observed phenomenon in preventing fungal penetration and colonization [41] and at all the interaction sites with AG K we detected densely stained cells enriched in phenolic compounds and callose surrounding the penetrating hyphae. Phenolic compounds not only form physical barriers for the pathogen, they are also known to have direct antimicrobial activities [42]. The granules present in the cortical cells adjacent to the penetration site as observed in this study are assumed to contain phenolic defence compounds synthesized by cauliflower in response to the attack by weak and non-aggressive *Rhizoctonia *isolates. At the reinforced cell walls, callose accumulation was also detected. Callose, a 1,3-β-glucan, may provide a physical barrier and has been described as a key component of penetration resistance in several plant-pathogen interactions [5,43,44]. Until now, strengthening of the cell wall in response to *Rhizoctonia *attack has only been reported for a non-pathogenic binucleate isolate of AG G. Jabaji-Hare et al. [17] described an increase in phenolic compounds, but not in callose during the interaction of bean and AG G, while Wolski et al. [45] showed an increase in both lignin and callose using a purified 1,3-α-glucan elicitor from AG G in potato sprouts. In this study, we reported the role of callose and phenolic compound deposition in the prevention of colonization of cauliflower by binucleate *Rhizoctonia *AG K and to a lesser extent by *R. solani *AG 3 and AG 5. The strong induction of cell wall fortifications with phenolic compounds and callose, leading to the arrest of the pathogen shortly after entrance of AG K implicates a state of high induced resistance. This might be linked with the biological control capacity ascribed to several non-pathogenic isolates of binucleate *Rhizoctonia *AGs [46]. The protection of several plant species by binucleate *Rhizoctonia *strains against infection by pathogenic *R. solani *isolates is a frequently studied topic [47-51]. Moreover, protection against fungal pathogens of other genera was observed [52-54]. The potential of the binucleate *Rhizoctonia *isolate of AG K used in this study as a biological control agent is still unclear and requires further research.

## Conclusion

In summary, we have shown that during the cauliflower-*Rhizoctonia *interaction different levels of disease severity are correlated with differences in infection rate, differences in host response and the ability to degrade pectic compounds. Highly pathogenic interactions were found for isolates of *R. solani *AG 1-1C, AG 2-1, AG 1-1B and AG 4 HGII and were characterized by a high infection rate in association with the absence of host responses and a strong pectin degradation. The slightly lower disease severity observed for AG 2-2 IIIb was due to a slower formation of infection cushions and the inability to degrade pectin. Furthermore, we detected that weak aggressive isolates of *R. solani *AG 3 and AG 5 and the non-pathogenic binucleate isolate of *Rhizoctonia *AG K entered the plant tissue both by the formation of infection cushions and by stomatal penetration, indicating all constitutive defence barriers present in cauliflower were defeated and differences in aggressiveness were caused by inducible defence responses. In addition, these defence responses were shown to include the deposition of phenolic compounds and callose of which different levels were detected at the interaction sites of the *Rhizoctonia *AGs, resulting in differences in disease severity.

## Authors' contributions

The studies were conceived and planned by JP and MH. JP carried out all experimental work and wrote the draft manuscript in consultation with MH. The manuscript was edited and prepared for submission by JP and MH. Both authors read and approved the final manuscript.
